# Clarifying space use concepts in ecology: Range vs. occurrence distributions

**DOI:** 10.1002/ecy.70300

**Published:** 2026-03-07

**Authors:** Jesse M. Alston, Christen H. Fleming, Michael J. Noonan, Marlee A. Tucker, Inês Silva, Cody Folta, Thomas S. B. Akre, Abdullahi H. Ali, Jerrold L. Belant, Dean Beyer, Niels Blaum, Katrin Böhning‐Gaese, Rogério Cunha de Paula, Jasja Dekker, Jonathan Drescher‐Lehman, Nina Farwig, Claudia Fichtel, Christina Fischer, Adam T. Ford, René Janssen, Florian Jeltsch, Peter M. Kappeler, Scott D. LaPoint, A. Catherine Markham, E. Patricia Medici, Ronaldo Gonçalves Morato, Ran Nathan, Kirk A. Olson, Bruce D. Patterson, Tyler R. Petroelje, Emiliano Esterci Ramalho, Sascha Rösner, Luiz Gustavo Rodrigues Oliveira‐Santos, Dana G. Schabo, Nuria Selva, Agnieszka Sergiel, Orr Spiegel, Wiebke Ullmann, Filip Zięba, Tomasz Zwijacz‐Kozica, George Wittemyer, William F. Fagan, Thomas Müller, Justin M. Calabrese

**Affiliations:** ^1^ Center for Advanced Systems Understanding, Helmholtz‐Zentrum Dresden‐Rossendorf Görlitz Germany; ^2^ School of Natural Resources and the Environment University of Arizona Tucson Arizona USA; ^3^ Department of Biology University of Central Florida Orlando Florida USA; ^4^ Conservation Ecology Center Smithsonian Conservation Biology Institute Front Royal Virginia USA; ^5^ Department of Biology University of British Columbia Okanagan Kelowna British Columbia Canada; ^6^ Department of Environmental Science Radboud University Nijmegen Netherlands; ^7^ Department of Biology University of Maryland College Park Maryland USA; ^8^ Hirola Conservation Programme Garissa Kenya; ^9^ Department of Fisheries and Wildlife Michigan State University East Lansing Michigan USA; ^10^ Michigan Department of Natural Resources Marquette Michigan USA; ^11^ Plant Ecology and Nature Conservation University of Potsdam Potsdam Germany; ^12^ Senckenberg Biodiversity and Climate Research Centre, Senckenberg Gesellschaft für Naturforschung Frankfurt Germany; ^13^ Department of Biological Sciences Goethe University Frankfurt Germany; ^14^ National Research Center for Carnivores Conservation, Chico Mendes Institute for the Conservation of Biodiversity Atibaia Brazil; ^15^ Dierecologie B.V Arnhem Netherlands; ^16^ Conservation Ecology, Department of Biology Philipps‐Universität Marburg Marburg Germany; ^17^ Behavioral Ecology and Sociobiology Unit German Primate Center Göttingen Germany; ^18^ Faunistics and Wildlife Conservation, Department of Agriculture, Ecotrophology, and Landscape Development Anhalt University of Applied Sciences Bernburg Germany; ^19^ Bionet Natuuronderzoek Stein Netherlands; ^20^ Black Rock Forest Cornwall New York USA; ^21^ Lamont‐Doherty Earth Observatory Columbia University Palisades New York USA; ^22^ Department of Anthropology Stony Brook University Stony Brook New York USA; ^23^ Lowland Tapir Conservation Initiative (LTCI), Instituto de Pesquisas Ecologicas (IPE) IUCN SSC Tapir Specialist Group (TSG) Campo Grande Brazil; ^24^ Movement Ecology Laboratory, Department of Ecology, Evolution and Behavior, Alexander Silberman Institute of Life Sciences The Hebrew University of Jerusalem, Edmond J. Safra Campus Jerusalem Israel; ^25^ Wildlife Conservation Society, Mongolia Program Ulaanbaatar Mongolia; ^26^ Integrative Research Center, Field Museum of Natural History Chicago Illinois USA; ^27^ Instituto de Desenvolvimento Sustentável Mamirauá Tefé Brazil; ^28^ Universidade Federal de Mato Grosso do Sul Campo Grande Brazil; ^29^ Institute of Nature Conservation Polish Academy of Sciences Krakow Poland; ^30^ Faculty of Life Sciences, School of Zoology Tel Aviv University Tel Aviv Israel; ^31^ Tatra National Park Zakopane Poland; ^32^ Department of Fish, Wildlife, and Conservation Biology Colorado State University Fort Collins Colorado USA; ^33^ Department of Ecological Modelling Helmholtz Centre for Environmental Research—UFZ Leipzig Germany; ^34^ Department of Geosciences TUD Dresden University of Technology Dresden Germany

**Keywords:** Brownian bridge movement model, home range, kernel density estimation, Kriging, movement ecology, movement model, space use, stochastic process models, utilization distribution

## Abstract

Quantifying animal movements is necessary for answering a wide array of research questions in ecology and conservation biology. Consequently, ecologists have made considerable efforts to identify the best way to estimate an animal's home range, and many methods of estimating home ranges have arisen over the past half a century. Most of these methods fall into two distinct categories of estimators that have only recently been described in statistical detail: those that measure range distributions (methods such as kernel density estimation that quantify the long‐run behavior of a movement process that features restricted space use) and those that measure occurrence distributions (methods such as Brownian bridge movement models and the Correlated Random Walk Library that quantify uncertainty in an animal movement path during a specific period of observation). In this paper, we use theory, simulations, and empirical analysis to demonstrate the importance of appropriately using these two categories of distributions and their estimators. Conflating range and occurrence distributions can have serious consequences for ecological inference and conservation practice. For example, in most situations, home ranges estimated using estimators of occurrence distributions are too small, and this problem is exacerbated by ongoing improvements in tracking technology that enable more frequent and more accurate data on animal movements. We encourage researchers to use estimators of range distributions to quantify home ranges and estimators of occurrence distributions to answer other questions in movement ecology, such as when and where an animal crossed a linear feature, visited a location of interest, or interacted with other animals.

## INTRODUCTION

Understanding how and why animals use the areas they inhabit is a core goal in the fields of ecology and conservation biology (Jeltsch et al., 2013; Nathan et al., 2008; Schick et al., 2008; Sutherland et al., 2013). The attributes of the areas where animals live shape their fitness, and knowledge of relationships between movement and fitness can inform our understanding of how animals interact with each other and their environments, as well as our ability to implement effective conservation interventions (Allen & Singh, 2016). For these reasons, the importance of quantifying space use was recognized early in the development of ecology and led to the concepts of “home ranges” and “utilization distributions.” The conceptual definition of home ranges provided by Burt (1943) is still the most widely cited and targeted for estimation. Burt defined an animal's home range as “…that area traversed by the individual in its normal activities of food gathering, mating, and caring for young. Occasional sallies outside the area, perhaps exploratory in nature, should not be considered as part of the home range.” Two and a half decades after Burt offered this definition, Jennrich and Turner (1969) coined the term “utilization distribution” as the probabilistic representation of a home range, providing a foundation for translating Burt's conceptual idea into statistical estimators that can be applied to animal location data (Horne et al., 2020). Together, these ideas have served as the foundation of research on animal movement and resource use over the past half a century.

Movement and resource use, however, are multifaceted aspects of animal behavior. Consequently, the home range concept has broadened substantially over time and there now exists a very large literature describing different approaches to home range estimation (Fieberg & Börger, 2012; Heit et al., 2021; Horne et al., 2020; Kie et al., 2010). Many of these approaches cluster around two distinct spatial probability distributions that arise from stochastic movement processes and can be estimated from animal location data. Fleming, Fagan, et al. (2015), and Fleming et al. (2016) referred to these as “range” and “occurrence” distributions, and others have begun to adopt this terminology (Horne et al., 2020; Keith et al., 2019; Scharf et al., 2018; Schlägel et al., 2019; Signer & Fieberg, 2021). Specifically, range distributions—which are most commonly estimated using minimum convex polygons (Dalke & Sime, 1938) and (autocorrelated) kernel density estimation (Fleming, Fagan, et al., 2015; Worton, 1989)—describe the long‐run behavior of a movement process that features restricted space use and are consistent with Burt's classic definition of the home range. In contrast, occurrence distributions—which are most commonly estimated using (dynamic) Brownian bridge movement models ([d]BBMMs; Horne et al., 2007; Kranstauber et al., 2012)—quantify uncertainty in the movement path of an individual during a period of observation and are not directly related to Burt's definition of the home range. If considering a random point along the sampled movement path as a prediction target, the occurrence distribution and range distribution are analogous to confidence intervals and prediction intervals, respectively, though both distributions are based on predictions. Both distributions can serve as estimation targets for which specific statistical estimators can be derived, but estimators of range distributions quantify fundamentally different phenomena than estimators of occurrence distributions: estimators of range distributions answer the question: “How much space is an animal likely to use over the long term?,” while estimators of occurrence distributions answer the question: “Where did an animal travel during a defined period of observation?” Although these questions may appear similar, range and occurrence distributions have very different biological and mathematical interpretations.

In this paper, we argue that range and occurrence distributions can serve as focal points around which to organize concepts, models, statistical estimators, and research questions. We use theoretical arguments, simulations, and empirical examples to demonstrate similarities and differences between these distributions, as well as consequences that can arise from conflating them (most commonly by using BBMMs to estimate home ranges). We then link estimators of range and occurrence distributions to the ecological questions each can answer.

## CONCEPTS AND DEFINITIONS

By explicitly separating the discrete‐time and often arbitrary sampling schedule from the underlying continuous‐time movement process, continuous‐time movement models offer a number of advantages over the more traditional approach of assuming a discrete‐time movement process (Kareiva & Shigesada, 1983; Langrock et al., 2012; Morales et al., 2004). These advantages include the ability to estimate scale‐invariant parameters, the ability to model movement using irregularly sampled data, and freedom from the assumption of serial independence among datapoints (Fleming et al., 2014b; Gurarie et al., 2017; Johnson et al., 2008). Defining movement in this way provides a framework that facilitates the derivation of rigorous statistical procedures for quantifying movement (Blackwell, 1997; Dunn & Gipson, 1977; Fleming, Fagan, et al., 2015; Hanks et al., 2015; Johnson et al., 2011; Michelot, Blackwell, & Matthiopoulos, 2019), including many non‐random behaviors such as migration, territoriality, patrolling, trap‐lining, collective movement, and habitat‐ or condition‐specific movement (e.g., Brennan et al., 2018; Moriarty et al., 2017; Papageorgiou & Farine, 2020; Péron et al., 2017; Sawyer et al., 2019). In this framework, we may consider an animal's trajectory collected from a telemetry movement track, with coordinates, $r\left(t\right)=\left(x\left(t\right),y\left(t\right)\right)$, to be a realization from a continuous‐time stochastic process, $R\left(t\right)$, that is observed at discrete times $t_{1},t_{2},t_{3},\cdots ,t_{n}$. From this realization, we can predict and estimate quantities related to the animal's movement patterns, conditional upon stochastic movement models that can be used to generate movement trajectories (Table 1). Some, but not all, stochastic movement models are range‐resident and feature finite coverage areas, in that their coverage areas—the smallest area in which a given percentage of an animal's locations is predicted to occur—converge to a finite extent as t approaches infinity (Table 1). This property manifests as an asymptote in semi‐variance function of a stochastic process as the time lag between observations of the process increases (Fleming et al., 2014a). Brownian motion (Einstein, 1905; Horne et al., 2007) and the integrated Ornstein–Uhlenbeck (IOU) process (Gurarie et al., 2017; Gurarie & Ovaskainen, 2011; Johnson et al., 2008) are endlessly diffusing processes and thus do not have finite coverage areas (i.e., their coverage areas expand forever). In contrast, models such as the Ornstein–Uhlenbeck (OU; Dunn & Gipson, 1977; Uhlenbeck & Ornstein, 1930) and Ornstein–Uhlenbeck foraging processes (OUF; Fleming et al., 2014a; Fleming, Subaşı, & Calabrese, 2015) are range‐resident and feature finite coverage areas. The OU and OUF processes can be thought of as range‐resident versions of Brownian motion and IOU processes, respectively. Another key distinction among movement models arises from the types of autocorrelation they can accommodate. Brownian motion and OU movement produce autocorrelated positions but uncorrelated velocities, while IOU and OUF movement produce both autocorrelated positions and autocorrelated velocities. In contrast, the independent and identically distributed (IID) process, while having a finite coverage area, produces—as the name implies—completely uncorrelated data. With these movement models in mind, we can define two key families of distributions that capture many (but not all) conceptions of “space use” in the ecological literature.

**TABLE 1:** Summary of stochastic processes that can currently be used to model animal movement.

Movement model	Position autocorrelation	Velocity autocorrelation	Range residency
IID	No	No	Yes
BM	Yes	No	No
OU	Yes	No	Yes
IOU	Yes	Yes	No
OUF	Yes	Yes	Yes

*Note*: These processes can feature positional autocorrelation, velocity autocorrelation, and/or range residency. The independent and identically distributed (IID) process can describe animal location data in which no autocorrelation is present. Brownian motion (BM) occurs in the limit of the Ornstein–Uhlenbeck (OU) process, when its positional autocorrelation time scale approaches infinity, while the integrated Ornstein–Uhlenbeck (IOU) process occurs when the positional autocorrelation time scale of the Ornstein–Uhlenbeck foraging (OUF) process approaches infinity. More detailed mathematical descriptions of these models can be found in Fleming et al., 2014a and Fleming, Subaşı, & Calabrese, 2015.

### The range distribution

Movement processes that feature finite coverage areas, including the IID, OU, and OUF processes, allow the possibility of a marginal probability density function $p\left(r\left(t\right)\right)=p_{R\left(t\right)}\left(r\left(t\right)\right)$ at each time t, which is the probability density of a location $r\left(t\right)$ at time t for the stochastic process $R\left(t\right)$, without conditioning on any previous or subsequent locations. In the most general sense, a range distribution is a marginal distribution focused on a particular time frame or suite of movement behaviors—possibly averaged over times or behavioral states—to enable predictions of an animal's locations in future periods. In other words, a range distribution describes the probability of an animal being in a location at a given time, taking into account all of the locations in a movement track simultaneously. The range distribution is simplest to define for stationary processes, which describe unchanging movement behaviors:

(1) $$p_{\text{range}}^{\text{stationary}}\left(r\right)=p_{R\left(t\right)}\left(r\right)=p\left(r\right),$$

for any time t. Non‐stationary processes, which describe movement behaviors that change over time (e.g., migrations, drifting home ranges), may further require an appropriate time average to weight the relevant marginal distributions (e.g., Fleming et al., 2018, S1). Because $p_{\text{range}}^{\text{stationary}}\left(r\right)$ denotes the relative frequencies of different locations of an animal in space, the range distribution provides a prediction of *space use*, in that 95% of future locations will fall within its 95% coverage area, so long as the underlying movement process does not change (a testable assumption; see Noonan et al., 2019).

Range distributions therefore quantify the long‐run (asymptotic) behavior of a movement process. They are generated by running a single realization of the movement process forward into the future while keeping movement behavior fixed. The coverage areas of the range distribution are not estimates of what space the animal has used during the observation period, but predictions of what space will eventually be used, given a sufficient amount of time for the movement process to continue. All else being equal, an IID process will very quickly enable accurate estimation of a range distribution as the sample size is increased, whereas highly autocorrelated processes such as OUF will take longer to enable accurate estimation of a range distribution, because their effective sample sizes will be much smaller than their nominal sample sizes. However, the autocorrelation in the resulting data contains information about the long‐run behavior of the process, and thus the *estimate* of the range distribution that accounts for autocorrelation in the data may contain a considerable amount of space that is not visited during a period of study. The range distribution corresponds closely to Burt's conceptual definition of home range because it quantifies the area that the animal typically uses, not including exploratory forays. Estimators of range distributions are thus the appropriate tools for answering the question: “How large is an animal's home range?”

Range distributions are part of a larger class of distributions called “limiting distributions” that describe the long‐run behavior of a stationary stochastic process. Although the value of limiting distributions for conceptualizing and characterizing animal movement processes has not been widely appreciated among movement ecologists, there has been some recent methodological development around this idea. Barnett and Moorcroft (2008) described a limiting distribution for resource selection models that can be used to inform mechanistic home range analysis (Moorcroft et al., 2006). Whitehead and Jonsen (2013) and Wilson et al. (2018) described limiting distributions of discrete‐space continuous‐time Markov chain processes that produce range distributions. Michelot, Gloaguen, et al. (2019) used the limiting distribution of a Langevin diffusion process to create utilization distributions, and also used Markov chain Monte Carlo sampling to construct a movement model whose limiting distribution is a known utilization distribution (Michelot, Blackwell, & Matthiopoulos, 2019).

When data are statistically independent, and thus consistent with the IID assumption, the range distribution can be estimated using a variety of methods including minimum convex polygons (MCPs), conventional kernel density estimation (KDE), and mechanistic home range analysis. For the autocorrelated data provided by modern technologies such as GPS and ATLAS (Kays et al., 2015; Nathan et al., 2022), the range distribution is most accurately estimated by autocorrelated Gaussian density estimation (Dunn & Gipson, 1977; Fleming et al., 2014b) if the home range is Gaussian, or autocorrelated kernel density estimation (AKDE; Fleming, Fagan, et al., 2015, Noonan et al., 2019) if the home range is not Gaussian. In other words, the estimation target of all of these estimators is the range distribution, but each estimator differs in the assumptions made about the data that underlie it. A given estimator must therefore be used only when the data are consistent with the movement model that underlies that estimator's assumptions (as is standard statistical practice).

Finally, we note that there is no dependence in the definition of the range distribution on the particular sampling regime chosen by an investigator. The range distribution is a property of the movement process that is independent of the sampling process. However, *estimators* of the range distribution are subject to a number of biases, some of which can be related to the sampling process (Silva et al., 2022). First, uncertainty in the size and shape of an estimated range distribution decreases in proportion to its “effective sample size,” which is approximately the number of times a focal animal moved far enough to cross its home range during an observation period. If the animal has not yet moved far enough to cross its range in an observation period, it is not possible to estimate an accurate range distribution, and if an animal has not crossed its range very many times in an observation period, it is difficult to accurately or precisely estimate range distributions. Uncertainty in estimates of range distributions can be in part represented by confidence bands around estimated coverage areas, as the coverage areas are often the key parameters of interest. Second, different estimators of range distributions may exhibit either positive or negative biases that decrease asymptotically as sampling duration increases. Third, estimators that assume IID data (e.g., conventional KDE, MCP, mechanistic home range analysis) tend to underestimate the size of home ranges when applied to autocorrelated tracking data by an extent that depends, all else equal, on the strength of autocorrelation in the sampled locations (Noonan et al., 2019). Again, this is not an inherent property of the range distribution per se, but, instead, results from using estimators for which a core assumption has been violated. As with any statistical procedure, violating a key assumption of a home range estimator can produce biased results.

### The occurrence distribution

Whereas range distributions are based on the marginal probability density functions, $p\left(r\left(t\right)\right)$, and can predict future locations, occurrence distributions are based on the conditional probability densities $p\left(r\left(t\right)|\text{data}\right)$ and are focused on interpolating the specific movement path that an animal took during an observation period and that connected a series of sampled locations. Such conditional distributions exist for all stochastic movement processes, even when those processes do not have finite coverage areas in the long run and do not describe range‐resident movement behaviors (e.g., Brownian motion and IOU movement). The simplest occurrence distribution that we can construct involves uniformly averaging these conditional densities over the observation period for times sampled between $t_{1}$ and $t_{n}$:

(2) $$p_{\text{occurrence}}\left(r\right)=\underset{\;\text{time}‐\text{average}\;}{\underbrace{\frac{1}{t_{n}-t_{1}}\int_{t_{1}}^{t_{n}}\;\mathrm{dt}}}\;p_{R\left(t\right)}\left(r|\text{data}\right),$$

which summarizes the full information on the unknown path contained in the time‐indexed conditional densities, $p\left(r\left(t\right)|\text{data}\right)$, into a single probability density, $p_{\text{occurrence}}\left(r\right)$. This corresponds to the prediction of an unknown location $r\left(t\right)$ at a random time t within the observation window, given the observed data. Missing observations are often skipped to avoid oversmoothing (e.g., Bedrosian et al., 2018; Coe et al., 2015; Sawyer et al., 2009), but weighting or imputation schemes that maintain a balance between detail and continuity (e.g., Buderman et al., 2016; Hooten & Johnson, 2017; Scharf et al., 2017) are available. In the limit of very coarse, uncorrelated data, and with some gap‐skipping heuristic applied, the occurrence distribution reduces to the empirical distribution of the data. This means that *there must be detectable autocorrelation in the movement process* for estimators of occurrence distributions to produce informative predictions of movement between observed locations—estimating an occurrence distribution using data that are so coarse as to be IID, or nearly so, will provide little information on unobserved locations.

Occurrence distributions quantify where an animal may have traveled during a given period of time, conditional on observed locations sampled within that period, and rely on autocorrelated movement models to interpolate the data between observed locations. They are not well estimated by the range distribution estimators outlined in the prior subsection, and occurrence distribution estimators have not been around nearly as long as range distribution estimators—the first estimator of occurrence distributions was introduced in the peer‐reviewed ecology literature less than 20 years ago (Horne et al., 2007). Occurrence distributions are calculated via model‐based time‐series Kriging, and Fleming et al. (2016) presented the general case. Earlier approaches to estimating occurrence distributions in the literature assumed specific movement models, such as Brownian motion (Horne et al., 2007; Kranstauber et al., 2012) or integrated Ornstein–Uhlenbeck motion (Johnson et al., 2011). Occurrence distributions exist for any autocorrelated movement process, however, whether or not the focal process assumes range‐resident movement. This means that the Brownian motion, IOU, OU, and OUF continuous‐time processes all admit occurrence distributions. For an IID process, the occurrence distribution is simply the empirical distribution, given some heuristic to account for gaps in the data. If large gaps are not skipped, then the effectively unconditioned predictions within those gaps will be as widespread as the underlying range distribution (infinite for Brownian and IOU motion) and will oversmooth the occurrence distribution into a large normal distribution.

Importantly, an estimator of occurrence distributions does not predict a movement path itself (because the movement path is a random variable, it is predicted rather than estimated), but uncertainty around that movement path—the movement path itself is best predicted by Kriging the most likely path, and occurrence distributions are the time‐average of that Kriging process' uncertainty distributions (i.e., the average predictive uncertainty around the most likely path); (Fleming et al., 2016). Estimates of the coverage areas of occurrence distributions are generated by considering all possible trajectories that are consistent with the data, weighted by their probability density. As the movement path of an animal becomes more finely and more accurately resolved, these coverage areas become smaller and smaller, eventually limiting to zero, even though the actual space used has not changed. *Estimated coverage areas of occurrence distributions therefore do not directly measure space use—even during the observation period—but are, instead, a reflection of our uncertainty regarding where an animal was located during an observation period*. In other words, if we have complete knowledge of the animal's locations during an observation period (i.e., infinite sampling rate and no location error), estimates of the occurrence distribution collapse to the animal's movement path and have zero area. The occurrence distribution is thus appropriate for answering questions such as “Where might an animal have traveled during an observation period?” and “What landscape features might an animal have visited along its movement path?”

**FIGURE 1:**
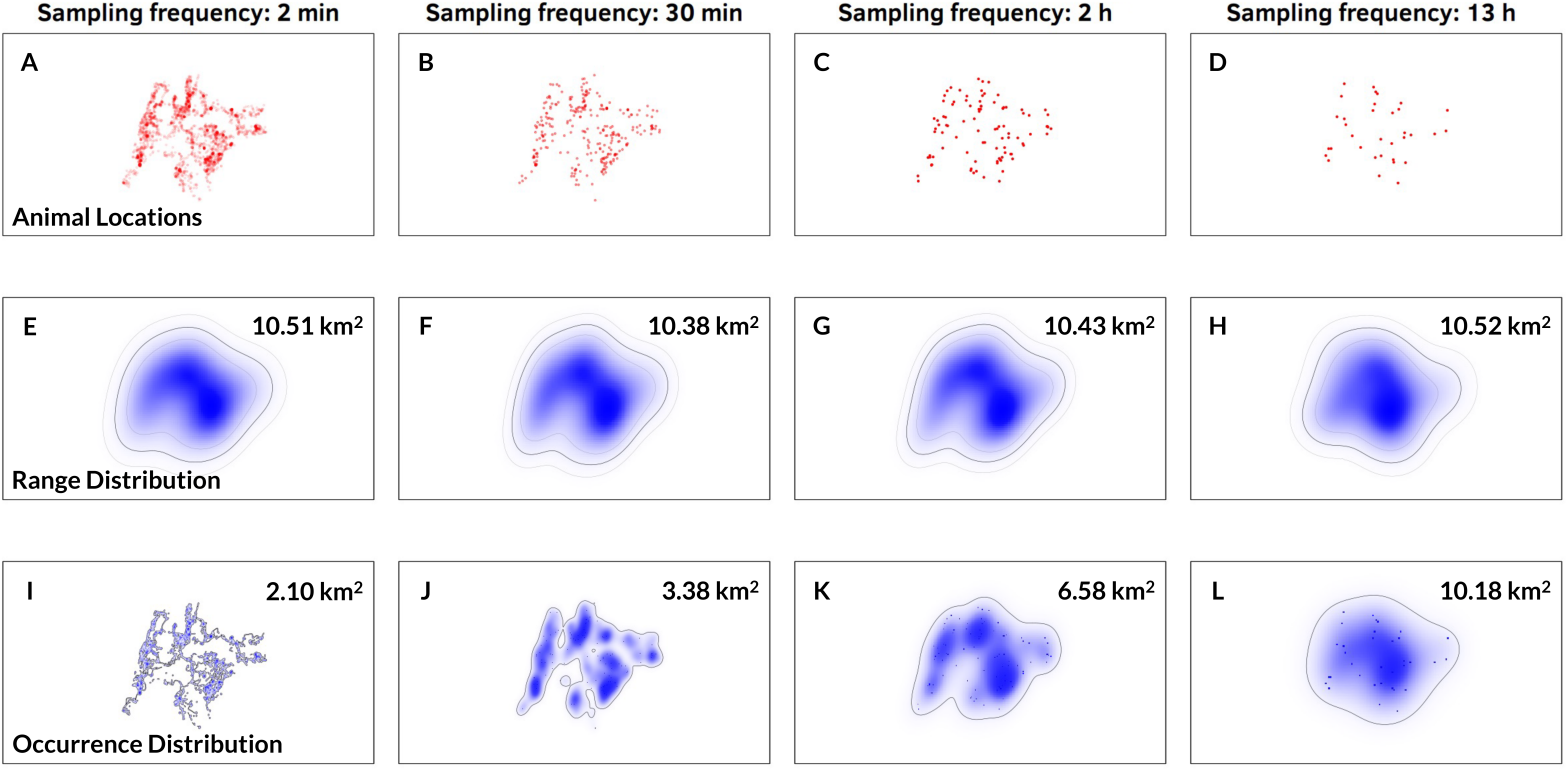
Demonstration of sampling dependence of estimated occurrence and range distributions using location data from a GPS‐tracked fisher (*Pekania pennanti*) from New York, USA. The fisher was tracked for 19 days at 2‐min intervals. The top row (panels A–D) features individual locations along the fisher's movement track as the movement track is progressively thinned from ca. 720 locations per day to ca. 2 locations per day. The second row (panels E–H) features estimated 95% coverage areas generated using autocorrelated kernel density estimation on these GPS locations. While the contours of the estimated range distribution change as the data are more finely resolved, the size of estimated coverage areas (labeled in the upper right of each panel) remains largely stable. The third row (panels I–L) features estimated 95% coverage areas by Kriging the same GPS location. In contrast with the estimated coverage areas of the range distributions, the size of estimated coverage areas of the occurrence distributions (also labeled in the upper right of each panel) decreases rapidly as the data are sampled more frequently and uncertainty in the fisher's movement path decreases. With the coarsest samples (panels H and L), estimated coverage areas for the two methods are similar. With the finest samples (panels E and I), however, the estimated coverage area of the occurrence distribution is only ca. 20% of the size of the estimated coverage area of the range distribution.

Transitioning from marginal distributions that are independent of specific observations to conditional distributions that are conditional upon preceding and subsequent observations has a dramatic effect on the meaning and operation of occurrence distributions. Range distributions and their constituent marginal distributions are parameters of the movement process that exist independently from the sampling process (though estimators of the range distribution may exhibit some sampling dependence). In contrast, occurrence distributions are conditional upon samples of data and are explicitly defined in terms of the sampling schedule. This means that a different sampling schedule applied to the same movement process will correctly yield a different occurrence distribution: all else equal, increasing the sampling rate will result in a narrower, more concentrated occurrence distribution. This happens because more frequent sampling more fully resolves the animal's true movement path, and thus uncertainty in the animal's locations decreases concomitantly. It is important to realize that this is not due to sampling‐dependent bias of estimators of occurrence distributions: occurrence estimators in the time‐series Kriging family, including the BBMM, can be unbiased. Instead, the uncertainty decreases because the estimation target itself (i.e., the occurrence distribution) is a function of the sampling schedule. Figure 1 shows this process occurring for data from a fisher (*Pekania pennanti*) tracked for 19 days in New York, USA, at a roughly 2‐min sampling interval.

While the occurrence distribution itself is a reflection of uncertainty in the unknown movement path, its parameter uncertainty has not been historically propagated. In fact, parameter uncertainty has only recently been propagated even among range distributions (Fleming & Calabrese, 2017), where the key parameters of interest are the coverage areas. However, for occurrence distributions, the key predictions of interest are expected values of spatial covariate functions. For instance, the expected value with respect to a vegetation cover index is the point prediction for the average of percent vegetation cover sampled along the unknown movement path (e.g., 70%). For these expected values, there is both the predictive uncertainty, reflecting the unknown movement path, and estimation uncertainty, reflecting the unknown movement parameters.

### Relationships between range and occurrence distributions

As detailed above, range and occurrence distributions are based on different biological and statistical definitions, have different interpretations and statistical estimators, and respond differently to variation in sampling schedules. We now consider two key limits defined by data amount and quality that highlight the conditions under which range and occurrence distributions either converge or diverge completely, and reiterate a conceptual difference between the two distributions.

#### Convergent limit: Infinite observation period

Given an infinite observation period, the occurrence distribution will converge to the range distribution for range‐resident movement processes. This happens because an animal visits more and more of its home range over time. Differences in estimated range and occurrence distributions can still arise from estimation error caused by gaps in data and measurement error. Decreasing location error and increasing the sampling rate will reduce this estimation error, but increasing the sampling rate will also slow down convergence because coverage areas of occurrence distributions limit to zero if the sampling rate is infinite while the observation period is finite.

#### Divergent limit: Infinite sampling rate

For the occurrence distribution of any realistic movement process that is continuous in both location and velocity, holding the sampling duration constant while increasing the sampling rate with either no location error or uncorrelated location error yields the limit:

(3) $$\lim_{\mathrm{dt}\to 0}p_{R\left(t\right)}\left(r|\text{data}\right)=\delta \left(r-r\left(t\right)\right),$$

where $\delta \left(r\right)$ is the Dirac delta function—a singular distribution with probability mass concentrated at r, and where $r\left(t\right)$ is the realized location. In other words, as sampling becomes finer and finer, the occurrence distribution collapses to the movement path with zero coverage area, even in the presence of (uncorrelated) location error. This limit is easiest to see in the case of a BBMM, where the width of the bridge is at its most proportional to dt. The range distribution is not subject to this limit and estimates of its coverage areas remain the same, though estimators of the range distribution may exhibit varying dependence on sample size. Increasing the rate of sampling results in increasingly strong autocorrelation in the data, so estimators of range distributions that do not account for this autocorrelation perform worse as sampling rate increases, all else being equal. Such estimators are increasingly negatively biased by increasing autocorrelation strength and will also limit to zero area. However, estimators of range distributions that properly model autocorrelation will be unaffected by this limit, and estimated coverage areas will remain consistent.

#### Extrapolation versus interpolation

Range and occurrence distributions can also be conceptualized in terms of two common categories of statistical inference: extrapolation and interpolation, respectively. Given a sample of tracking data of finite duration, an estimate of the range distribution represents an *extrapolation* of the long‐run behavior of the movement process, as inferred from the data, and quantifies the variance of the movement process. In contrast, an estimate of the occurrence distribution *interpolates* within the observation period, conditional on the data and an autocorrelated movement model, and quantifies uncertainty in the interpolation. This is why the general framework for estimation of occurrence distributions is based on Kriging, which is a statistically optimal method of model‐based interpolation (Fleming et al., 2016).

To illustrate this more concretely, consider the differences in how cross‐validation of estimators of home range distributions and cross‐validation of estimators of occurrence distributions are performed. If an estimator accurately quantifies the areas of home ranges (sensu Burt, 1943), 95% coverage areas of home ranges generated over some observation periods $T_{1}$ should contain, on average, 95% of those same animals' locations over subsequent observation period $T_{2}$, provided those animals' movement behaviors do not meaningfully change between training ($T_{1}$) and test ($T_{2}$) sets, and provided that $T_{1}$ and $T_{2}$ begin far enough apart to be uncorrelated. If 95% coverage areas of home ranges consistently include more than 95% of the locations in test sets, then estimators are overestimating the sizes of home ranges; if those coverage areas consistently include fewer than 95% of locations in test sets, then estimators are underestimating the sizes of home ranges. Similarly, 50% coverage areas generated over $T_{1}$ should contain 50% of animals' locations, on average, over $T_{2}$. In other words, the relevant test set for an estimator of range distributions is a set of locations sampled from an animal's movements *in the future*, extrapolated from past locations (half‐sample cross‐validation; e.g., Noonan et al., 2019). Cross‐validation of estimators of occurrence distributions should operate differently. If an estimator quantifies occurrence distributions accurately, 95% coverage areas estimated using locations sampled from $T_{1}$ should contain 95% of those animals' unobserved locations occurring within $T_{1}$. In other words, the relevant test set for an estimator of occurrence distributions is holdout data from within an observation period (and not a subsequent period).

## SIMULATED EXAMPLES

The two limits described above are crucial for understanding the differences between occurrence and range distributions. We now demonstrate the importance of these limits with both simulated and real data. For the simulated data, we can specifically model processes where both types of distributions exist: processes that are (1) autocorrelated (so that estimators of the occurrence distribution can interpolate the data) and (2) range‐resident (so that a range distribution exists). Simulation also allows us to set known parameters for the home range (range distribution). We can then manipulate the sampling schedule of the simulated processes to explore the effects of sampling rate and sampling duration on the sizes of estimated range and occurrence distributions. To do this, we simulated movement paths from an OUF process while varying the sampling rate and sampling duration systematically to illustrate differences between estimates provided by estimators of range and occurrence distributions. For each dataset, we estimated the range distribution via autocorrelated Gaussian density estimation conditioned on a fitted OUF model with daily autocorrelation timescales. Similarly, we estimated the occurrence distribution for each dataset by Kriging with an OUF model with daily autocorrelation timescales. The process that generated the simulated data exactly matches the statistical assumptions of these estimators, so any differences in size between estimates of the two distributions reflect differences in the distributions rather than differences in data used to estimate them.

**FIGURE 2:**
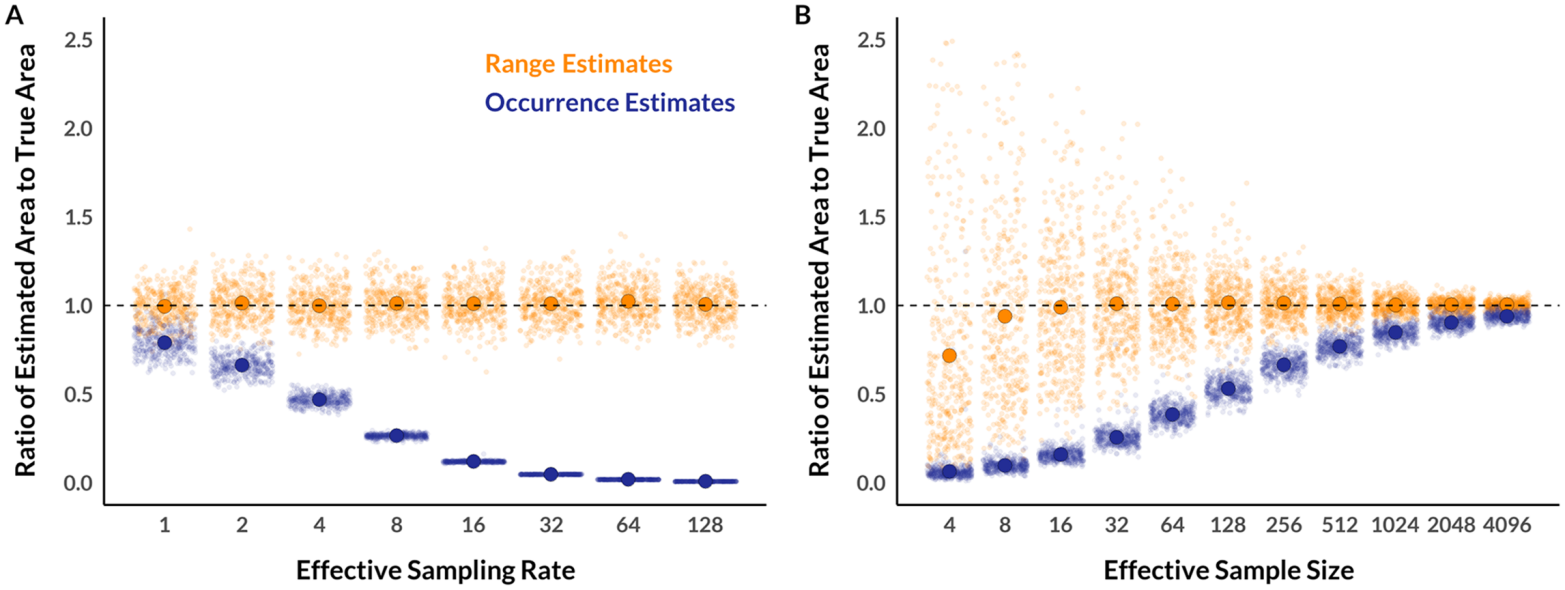
Bias in estimated home range size provided by coverage areas of range (orange) and occurrence (indigo) distributions estimated using data with different sampling rates (i.e., GPS fixes per range crossing; panel A) and effective sample sizes (i.e., number of range crossings in a dataset; panel B). A point at 1 indicates that home range size was estimated correctly for a simulated movement track with a known home range size. Small points represent a single simulation result (jittered on the *x*‐axis to ease visualization), while larger points represent the mean simulation result among 400 replicates. Divergence of estimated range distributions from the line at 1 at small durations of sampling arise from known patterns of bias that can be improved by bootstrapping (Fleming et al., 2019), while bootstrapping does little to change the size of estimated occurrence distributions.

Figure 2A shows the estimated coverage areas of the occurrence distribution decreasing in size as the sampling rate increases from one observation per day to 128 observations per day. Note that the estimated 95% coverage areas of the occurrence distribution start substantially smaller than the estimated 95% coverage areas of the range distribution, because the sampling duration is finite (256 days, in this case), and then rapidly collapses to zero as the sampling rate increases. Ongoing technological advances that facilitate ever finer and more accurate location sampling are driving movement studies closer to the limit where estimated occurrence distributions collapse to zero area. It is therefore inevitable that the differences between range and occurrence distributions will become more obvious in the future, even though these distributions have been frequently conflated in the past.

Figure 2B shows the estimated coverage areas of the occurrence distribution increasing as the sampling duration increases from 4 to 4096 days. Again, the estimated 95% coverage areas of the occurrence distribution are still substantially smaller than the estimated 95% coverage areas of the range distribution even when the effective sample size (i.e., the number of range crossings) is >4000, and rapidly collapse to zero as the duration of the observation period decreases. This is a major real‐world problem because it can take weeks or months on average for an animal to cross its range (depending on species), and the lifespan of tracking devices (or even animals) is unlikely to enable an effective sample size of anywhere near 4000 (i.e., 11 years with daily range crossings). This demonstrates that while occurrence distributions tend toward range distributions as the sampling duration increases, using an estimator of occurrence distributions to quantify the size of home ranges will yield a substantial underestimate unless data are collected for a very long period of time—periods of time that are likely logistically and/or biologically impossible. Although technological advances are increasing the battery lifespan of animal tracking devices, and thus the potential duration of animal tracking studies, the duration of tracking data for an individual animal is often limited in practice by mortality or equipment failure. Estimators of occurrence distributions will therefore tend to provide estimated home ranges that are substantially smaller than the true size of home ranges in most real‐world situations.

## EMPIRICAL EXAMPLES

**FIGURE 3:**
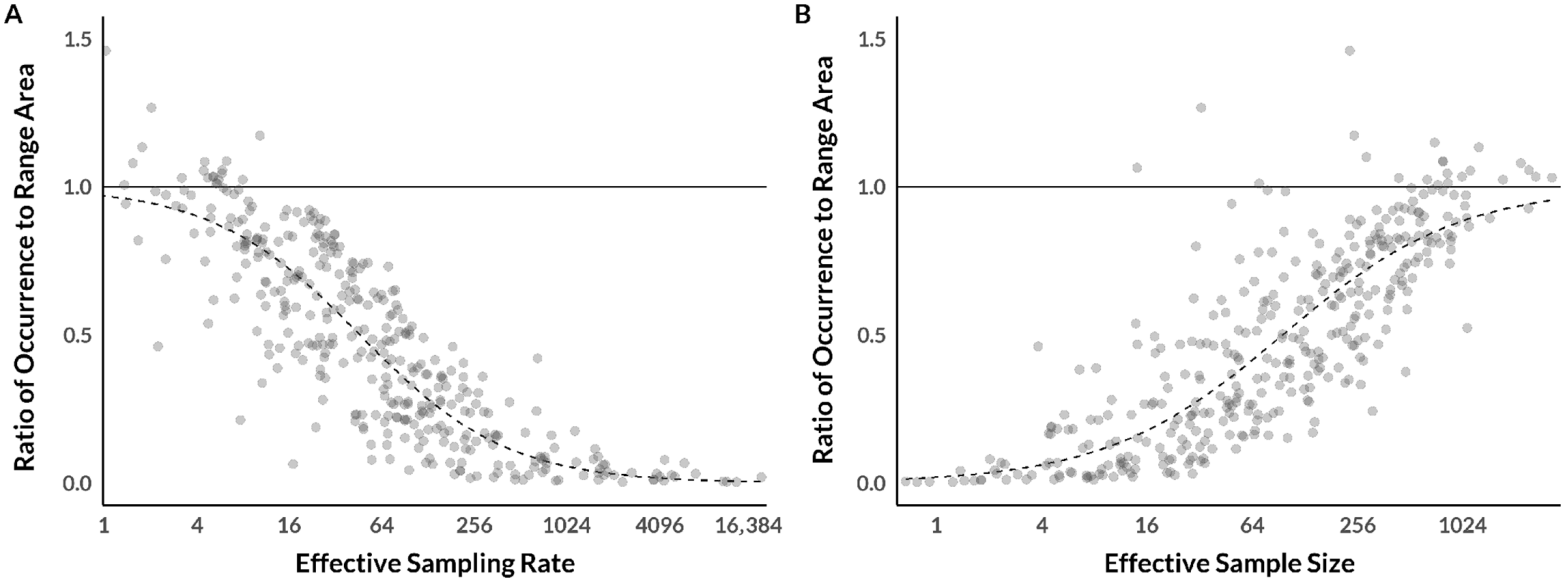
The ratio of the size of estimated 95% coverage areas of occurrence distributions to the size of estimated 95% coverage areas of range distributions for a dataset containing 369 individuals across 27 species, as a function of the effective sampling rate (i.e., GPS fixes per range crossing; panel A) and effective sample size (i.e., number of range crossings in a dataset; panel B). Points represent individual animals, while dashed lines represent regressions demonstrating the overall trend. Solid horizontal lines indicate a ratio of 1:1, where estimated range and occurrence distributions are the same size. Distance below the solid line indicates the extent to which estimators of occurrence distributions are negatively biased in their estimates of home range size.

Using empirical data, we now show how profoundly estimates of range and occurrence distributions can diverge in real‐world datasets. As outlined above, this happens when the data are sampled frequently enough that the occurrence distribution collapses toward the movement path and for long enough that estimating the range distribution is possible. Such datasets are already common and their availability will only increase as tracking technology improves (Gupte et al., 2022; Kays et al., 2015; Nathan et al., 2022). Using a dataset of 369 individual animals across 27 species (Noonan et al., 2019), we estimated both the range and occurrence distributions for each animal. We estimated the range distribution via autocorrelated kernel density estimation (AKDE) conditioned on a fitted movement model according to the workflow described in Silva et al. (2022). In short, we used variogram analysis (Fleming et al., 2014a) to ensure that animals were range‐resident, fit and selected an autocorrelated movement model that best described the animal's movements using perturbative hybrid residual maximum likelihood (*phREML*; Fleming et al., 2019) and Akaike's information criterion corrected for small sample sizes (AICc; Hurvich & Tsai, 1989), and estimated weighted AKDE utilization distributions for each animal (Fleming et al., 2018) using the ctmm R package (v0.6.2; Calabrese et al., 2016) in the R statistical software environment (v3.6.2; R Core Team, 2020). We estimated the occurrence distribution for each animal based on Kriging (Fleming et al., 2016) with the same movement model used for the corresponding AKDE estimate. Figure 3 shows that the estimated 95% coverage areas of occurrence distributions are smaller than estimated 95% coverage areas of range distributions for the vast majority of individuals in the dataset (and usually much smaller). This occurs because the effective sample size of real‐world datasets—which typically depends more on the duration of sampling than on the frequency of sampling—is rarely large enough to approach the theoretical limit where the occurrence distribution would converge with the range distribution.

The smaller coverage areas of estimated occurrence distributions compared to estimated range distributions consistently lead to underestimation of the size of animal home ranges. To illustrate this, we performed half‐sample cross‐validation on the same animal location dataset. We subset data from each individual animal into halves, used the first half of the data to generate estimates of range distributions (via AKDE) and occurrence distributions (via Kriging), and then used the second half to assess the percentage of future animal locations that were within the estimated range and occurrence distributions. All data fit the assumptions of range‐resident animals with movement processes that remained consistent between the two halves. We then fit regression lines (linear for AKDE estimates, logistic for Kriged estimates) for the influence of effective sampling rate (roughly the number of GPS locations per range crossing) and effective sample size on the percentage of locations in the test set that fell within estimates generated using the training set.

**FIGURE 4:**
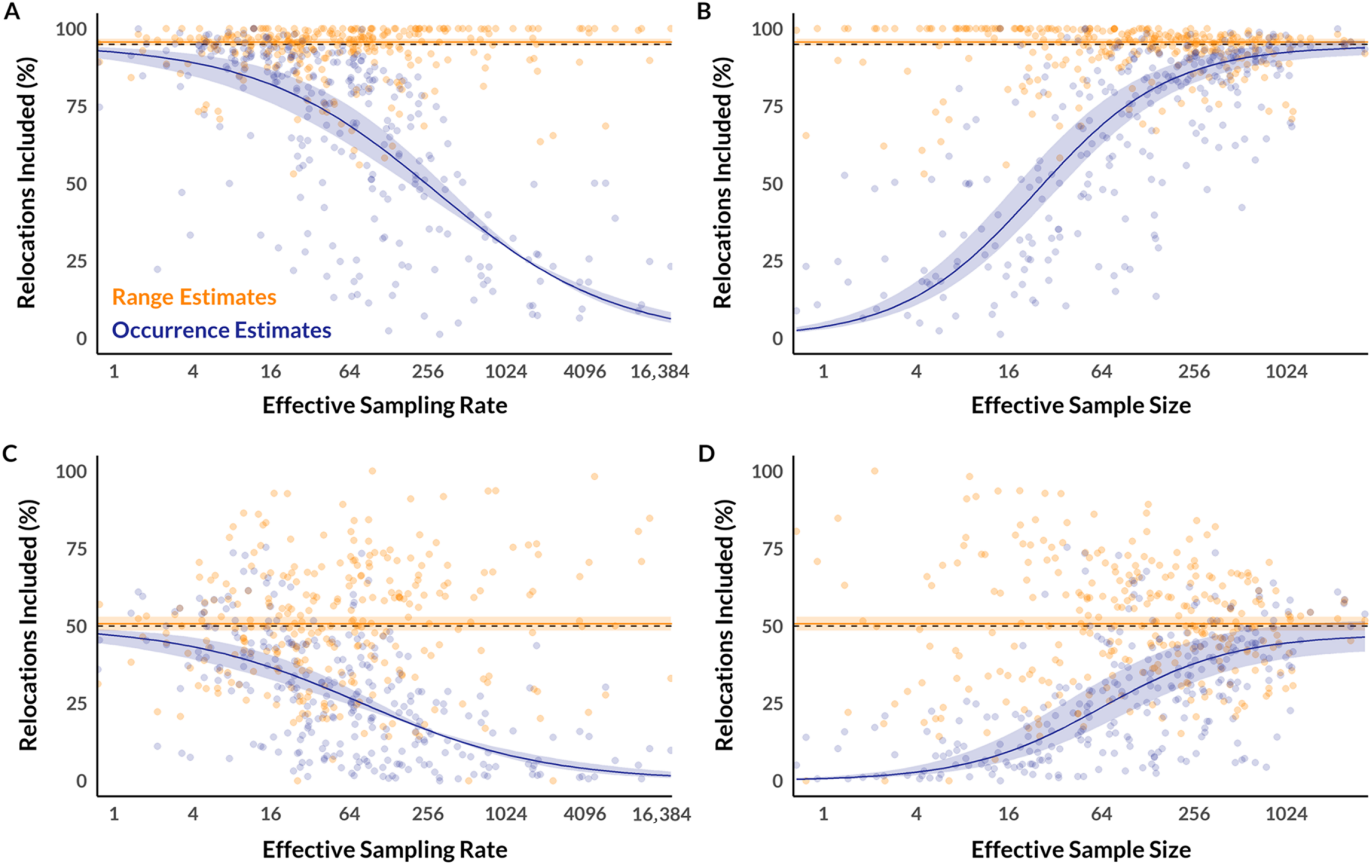
Half‐sample cross‐validation of estimated range and occurrence distributions. Points represent the percentage of locations from the second half of the data (test set) included in home ranges estimated using the first half of the data (training set). Orange points represent range (autocorrelated Kernel density estimation) estimates, while indigo points represent occurrence (Kriging) estimates. The dashed line represents the target 95% (top row) or 50% (bottom row) quantile, while the solid line represents a regression model fit to the cross‐validation results with shading to indicate the 95% confidence interval. The left column demonstrates the influence of effective sampling rate on cross‐validation results, while the right column demonstrates the influence of effective sample size on cross‐validation results. On average, estimated range distributions contain roughly the correct percentage of relocations, and this remains true across all effective sampling rates and effective sample sizes. Estimated occurrence distributions, however, tend to contain too few relocations, and this problem is exacerbated at high effective sampling rates and low effective sample sizes.

As these results show (Figure 4), estimated home ranges produced by estimators of occurrence distributions do not merely fit the data more tightly—they inaccurately represent the size of home ranges. Estimated home ranges produced by estimators of occurrence distributions are nearly always too small, and this negative bias is exacerbated at high sampling rates and low effective sample sizes. This is not merely a hypothetical problem, nor is it only an issue that will arise in the future as technology continues to improve. Instead, it is pervasive in the animal movement data that wildlife biologists currently collect and analyze (Noonan et al., 2019; Noonan et al., 2020).

## DISCUSSION

Ecologists often conflate estimators of occurrence distributions with estimators of range distributions, a much older and more familiar class of statistical tools. The first widely used estimators of occurrence distributions (Horne et al., 2007; Johnson et al., 2011) were landmark advances in movement ecology and enabled more statistically rigorous analyses of many research questions related to animal movement. Nevertheless, although they have been widely used in movement ecology, their novelty and unique statistical properties have still gone unrecognized all too often. As we have demonstrated, estimators of range and occurrence distributions have radically different properties and should therefore be used for different purposes (Table 2). Ecologists and conservation biologists should use estimators of range distributions to estimate home ranges, and estimators of occurrence distributions to answer other questions, such as the following: How likely is it that an animal visited a location of interest (Johnson et al., 2011; Noonan et al., 2018; Pagès et al., 2019; Scharf et al., 2017)? Where might an animal have crossed a linear feature (Find'o et al., 2018; Hooker et al., 2020; Zeller et al., 2018)? When and where could two individual animals have interacted (Schlägel et al., 2019)? Which areas of a landscape contain high‐priority resources (e.g., migratory corridors, stopover sites, and feeding grounds; Johnson et al., 2011, Sawyer et al., 2009, 2019)?

**TABLE 2:** Summary of the primary distinctions between range and occurrence distributions.

	Range distribution	Occurrence distribution
Distribution type	Marginal	Conditional
Finite coverage area	Arises only when the stochastic movement process being modeled has a finite coverage area	Arises when the sampling rate is finite, regardless of whether the stochastic movement process being modeled has a finite coverage area
Statistical operation	Extrapolation (Over what area is an animal likely to range in the future?)	Interpolation (At which locations might an animal have occurred in the past?)
Sampling dependence	No (If its statistical assumptions are met, an estimator of range distributions will estimate stable coverage areas even as a movement track is sampled more frequently)	Yes (If its statistical assumptions are met, an estimator of occurrence distributions will estimate smaller coverage areas as a movement track is sampled more frequently)
Appropriate questions & examples	How large is an animal's home range?^1–3^ How do environmental and social factors influence animal area requirements?^4–6^ Where are individual animals likely to interact over time?^7^ What area is available to an animal in studies of third‐order habitat selection?^8–10^	How likely is it that an animal visited a location of interest?^11–14^ Where might an animal have crossed a linear feature?^15–17^ When and where could two individual animals have interacted?^18^ In which areas of a landscape did an animal visit high‐priority resources (e.g., migratory corridors, stopover sites, or feeding grounds)?^19–21^

*Note*: Superscript numbers denote the following examples in the scientific literature: (1) Meyer et al., 2021, (2) Mirski et al., 2021, (3) Noonan et al., 2020, (4) Butler et al., 2022, (5) Dickie et al., 2022, (6) Snider et al., 2021, (7) Noonan et al., 2021, (8) Bista et al., 2023, (9) Ford et al., 2023, (10) Gill et al., 2023, (11) Johnson et al., 2011, (12) Noonan et al., 2018, (13) Pagès et al., 2019, (14) Scharf et al., 2017, (15) Find'o et al., 2018, (16) Hooker et al., 2020, (17) Zeller et al., 2018, (18) Schlägel et al., 2019, (19) Johnson et al., 2011, (20) Sawyer et al., 2009, (21) Sawyer et al., 2019.

**FIGURE 5:**
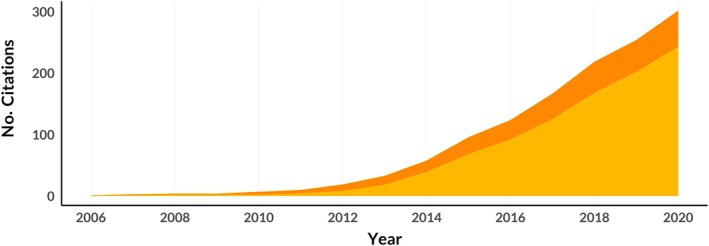
Number of peer‐reviewed journal articles from 2006 to 2020 that either used Brownian bridge movement models (BBMMs) to estimate the size of animal home ranges (light orange) or labeled BBMMs as a home range estimator (dark orange). Although the BBMM is an estimator of occurrence distributions and therefore poorly suited for estimating home ranges, the use of BBMMs to estimate home ranges is growing rapidly.

Estimation of animal home ranges is foremost among our concerns on the conflation of range and occurrence distributions—estimators of occurrence distributions consistently and substantially underestimate the size of home ranges under a broad array of real‐world conditions (Figures 3 and 4). In recent years, there has been a slow but steady drift in preference among wildlife biologists toward estimators that fit more tightly to animal location data (Figure 5; Crane et al., 2021, Laver & Kelly, 2008, Walter et al., 2015). We believe that this preference has largely been driven by the intuitive notion that areas within estimated home ranges where an animal does not travel during a study are not actually “used” (Cumming & Cornélis, 2012; Getz et al., 2007; Kie, 2013; Walter et al., 2015). This preference can be observed in the transition over time from home range estimates using minimum convex polygons to local convex hull (LoCoH; Getz et al., 2007, Getz & Wilmers, 2004) to time local convex hull (T‐LoCoH Lyons et al., 2013) methods, an emphasis on KDE bandwidth optimizers that fit tightly to location data (Cohen et al., 2018; Downs & Horner, 2008; Kie, 2013), and most recently, rapid growth in use of BBMMs to estimate home ranges (Figure 5). Cross‐validation frameworks that seek to backtest estimator performance (e.g., Getz & Wilmers, 2004; Kie, 2013; Silva et al., 2020; Walter et al., 2015), which are appropriate for assessing estimators of occurrence distributions but not estimators of range distributions, have also provided a false impression that range estimators that generate smaller home ranges perform better. While understandable, seeking home range estimates that fit tightly to an animal's past locations adheres neither to Burt's widely cited definition nor the mathematical properties underlying the range distribution. Researchers typically aim to capture the amount of space an animal needs to survive and reproduce in the long run, not simply the level of uncertainty in an animal's movement path during an observation period limited by study design, technology, or animal mortality. When comparing multiple estimators, smaller home range estimates are only better if coverage areas provide predictions of future animal locations that are at least as unbiased as the estimator that produces the larger estimate.

The most common estimator of occurrence distributions used to estimate home ranges is the BBMM (Horne et al., 2007), which has even been championed as a “third generation home range estimator” due to its ability to account for some autocorrelation in tracking data (Walter et al., 2015). Figure 5 shows the cumulative number of peer‐reviewed journal articles between 2007 (when the BBMM was introduced to ecologists) and 2020 that either label it a home range estimator or use it to estimate the sizes of animal home ranges. However, Fleming et al. (2016) formally proved that the BBMM is an estimator of the occurrence distribution (rather than the range distribution) that arises as a special case of the more general time‐series Kriging family of estimators of occurrence distributions. Specifically, Kriging a movement track conditional on a Brownian motion movement model is equivalent to the BBMM. Beyond being an estimator of occurrence distributions, and thus only suited to the task of home range estimation in the (unrealistic) infinite data limit, the BBMM is also based on an endlessly diffusing Brownian motion process, which does not have a finite coverage area. Note that this is not a critique of the validity of the BBMM as an analytical tool per se. Other estimators of occurrence distributions, such as time‐series Kriging conditional on an OUF movement process, are also inappropriate for estimating the sizes of home ranges (as demonstrated in Figures 1, 2, 3), and BBMMs are the best tool currently available for quantifying where an animal might have been during an observation period if the Brownian motion model accurately characterizes the animal's movement process. Furthermore, when BBMMs were developed, the issue of underestimation of home ranges as outlined above was not as apparent because animal location data were coarser than they are today. However, as animal tracking technology improves and the resolution of datasets increases, the discrepancy between the coverage areas that BBMMs estimate and proper coverage areas for range distributions will continue to widen, and repeated studies of the same species with improved technology will lead to progressively smaller estimates of home ranges if these estimates are generated using estimators of occurrence distributions like BBMMs.

Using estimators of occurrence distributions to quantify home ranges can therefore have pernicious consequences for area‐based conservation strategies and for ecological inference. For example, many protected areas (e.g., the Attwater Prairie Chicken National Wildlife Refuge, Kirtland's Warbler Wildlife Management Area, and the National Key Deer Refuge in the USA, the Arawale National Reserve in Kenya, and the Blackbuck Conservation Area in Nepal) are designed to protect a focal species. For these protected areas, understanding how much space is required to maintain populations that are viable over the long term is vital for ensuring their effectiveness (Brashares et al., 2001; Pe'er et al., 2014). When protected areas are too small relative to their focal species' area requirements, the probability of population declines or extirpation increases significantly (Brashares et al., 2001; Gaston et al., 2008). Undersized protected areas also force a greater proportion of individuals into human–wildlife conflict at protected area boundaries (van Eeden et al., 2018) as relatively more animals must forage outside of protected areas (Farhadinia et al., 2018). It is thus critical that policy actions be well informed on area requirements of target species. To ensure that protected areas are adequately sized, estimates of the area required for an individual of a given species to persist and reproduce are often quantified via home range analysis (Martins et al., 2013; Rechetelo et al., 2016; Tédonzong et al., 2018). Because estimators of occurrence distributions underestimate the future area traversed by GPS‐tracked animals (often dramatically so; Figure 4), using estimators of occurrence distributions to estimate area requirements can result in protected areas that do not accomplish their intended purpose.

Conflating range and occurrence distributions when quantifying space use is also dangerous in its implications for basic inference in ecology. For example, the distinction between range and occurrence distributions is particularly salient for studies of resource use and selection by animals. Resource selection is generally studied using resource selection functions—which compare environmental covariates at the locations where animals were present (i.e., “used” locations) to covariates at locations taken from an area assumed to be available for selection (i.e., “available” locations; Manly et al., 2007)—or resource utilization functions, which compare intensity of use among an animal's used locations (Marzluff et al., 2004; Millspaugh et al., 2006). Estimators of range distributions are appropriate tools for quantifying *availability* for resource *selection* functions, because they characterize the area an animal is likely to travel over the long term. In contrast, estimators of occurrence distributions are appropriate for quantifying resource *use* in resource *utilization* functions, because they characterize an animal's likely presence on the landscape during a study period. In practice, ecologists typically (and correctly) use estimators of range distributions to sample availability in resource selection functions, but often use estimators of range distributions rather than occurrence distributions to quantify habitat use in resource utilization functions (e.g., Berry et al., 2019; Johnston et al., 2020; Koizumi & Derocher, 2019; Prince et al., 2016; Winder et al., 2017; but see Eckrich et al., 2020; Marques et al., 2020; Petroelje et al., 2021). This may be because the initial papers on resource utilization functions (Marzluff et al., 2004; Millspaugh et al., 2006) used estimators of range distributions to generate utilization distributions (understandable because estimators of range distributions were the only tools available at the time—estimators of occurrence distributions had not been popularized yet). Nevertheless, an increasing number of methods for estimating occurrence distributions have become available over the past two decades (Fleming et al., 2016; Horne et al., 2007; Johnson et al., 2011), and we encourage ecologists to use these estimators of occurrence distributions—rather than range distributions—to quantify resource use in resource utilization functions.

Tracking data can and should be a resource for informing our understanding of animal ecology. Although we are now better positioned than ever to use tracking data to estimate different aspects of space use by animals, capturing maximal value from tracking data requires ecologists to understand and use the most rigorous statistical tools and definitions currently available. In this paper, we have highlighted the distinction between range and occurrence distributions, delineated the conditions under which they will behave similarly and differently, mapped ecological questions and statistical estimators to each distribution, and demonstrated the negative consequences of continuing to conflate these two distributions. Estimators of both range and occurrence distributions are readily available today in free and open source software (Calabrese et al., 2016; Calenge, 2006; Johnson et al., 2008; Nielson et al., 2013; Signer et al., 2019), and we encourage readers to explore the important distinction between range and occurrence distributions themselves.

## AUTHOR CONTRIBUTIONS

Justin M. Calabrese conceived the idea; Christen H. Fleming developed the mathematical theory and arguments; Jesse M. Alston, Justin M. Calabrese, Christen H. Fleming, Michael J. Noonan, and Inês Silva conducted the analyses; and Jesse M. Alston and Justin M. Calabrese led the writing of the manuscript. Thomas S. B. Akre, Abdullahi H. Ali, Jerrold L. Belant, Dean Beyer, Niels Blaum, Katrin Böhning‐Gaese, Rogério Cunha de Paula, Jasja Dekker, Jonathan Drescher‐Lehman, Nina Farwig, Christen H. Fleming, Adam T. Ford, René Janssen, Florian Jeltsch, Peter M. Kappeler, Scott D. LaPoint, A. Catherine Markham, E. Patricia Medici, Ronaldo Gonçalves Morato, Ran Nathan, Kirk A. Olson, Bruce D. Patterson, Tyler R. Petroelje, Emiliano Esterci Ramalho, Sascha Rösner, Luiz Gustavo Rodrigues Oliveira‐Santos, Dana G. Schabo, Nuria Selva, Agnieszka Sergie, Orr Spiegel, Wiebke Ullmann, Filip Zięba, and Tomasz Zwijacz‐Kozica contributed data for empirical analyses. All authors contributed critically to drafts of the manuscript and gave final approval for publication.

## CONFLICT OF INTEREST STATEMENT

The authors declare no conflicts of interest.

## Supporting information


Appendix S1.

## Data Availability

Tracking data on *Aepyceros melampus*, *Beatragus hunteri*, *Bycanistes bucinator*, *Cerdocyon thous*, *Eulemur rufifrons*, *Glyptemys insculpta*, *Gyps coprotheres*, *Madoqua guentheri*, *Ovis canadensis*, *Propithecus verreauxi*, *Sus scrofa*, and *Ursus arctos* are available in Dryad (Noonan et al., 2018; https://doi.org/10.5061/dryad.v5051j2), as are data from *Procapra gutturosa* (Fleming et al., 2014a; https://doi.org/10.5061/dryad.45157). Data on *Panthera onca* are available in *Ecology* (Morato et al., 2018; https://doi.org/10.1002/ecy.2379). Additional data are publicly archived in the Movebank repository, with identifiers provided in Appendix S1: Table S1. All other data and code required to reproduce results presented in this paper are available in Zenodo (Alston, 2025; https://doi.org/10.5281/zenodo.17676444).

## References

[ecy70300-bib-0001] Allen, A. M. , and N. J. Singh . 2016. “Linking Movement Ecology with Wildlife Management and Conservation.” Frontiers in Ecology and Evolution 3: 155.

[ecy70300-bib-0002] Alston, J. 2025. “Code and Metadata for: Clarifying Space use Concepts in Ecology: Range vs. Occurrence Distributions.” Dataset. Zenodo. 10.5281/zenodo.17676444. PMC12966954 41793168

[ecy70300-bib-0003] Barnett, A. H. , and P. R. Moorcroft . 2008. “Analytic Steady‐State Space Use Patterns and Rapid Computations in Mechanistic Home Range Analysis.” Journal of Mathematical Biology 57: 139–159. 18064464 10.1007/s00285-007-0149-8

[ecy70300-bib-0004] Bedrosian, B. E. , R. Domenech , A. Shreading , M. M. Hayes , T. L. Booms , and C. R. Barger . 2018. “Migration Corridors of Adult Golden Eagles Originating in Northwestern North America.” PLoS One 13: e0205204. 30462652 10.1371/journal.pone.0205204 PMC6248900

[ecy70300-bib-0005] Berry, L. E. , F. A. L'Hotellier , A. Carter , L. Kemp , R. P. Kavanagh , and D. A. Roshier . 2019. “Patterns of Habitat Use by Three Threatened Mammals 10 Years after Reintroduction into a Fenced Reserve Free of Introduced Predators.” Biological Conservation 230: 1–9.

[ecy70300-bib-0006] Bista, D. , G. S. Baxter , N. J. Hudson , and P. J. Murray . 2023. “Seasonal Resource Selection of an Arboreal Habitat Specialist in a Human‐Dominated Landscape: A Case Study Using Red Panda.” Current Zoology 69: 1–11. 36974152 10.1093/cz/zoac014 PMC10039176

[ecy70300-bib-0007] Blackwell, P. G. 1997. “Random Diffusion Models for Animal Movement.” Ecological Modelling 100: 87–102.

[ecy70300-bib-0008] Brashares, J. S. , P. Arcese , and M. K. Sam . 2001. “Human Demography and Reserve Size Predict Wildlife Extinction in West Africa.” Proceedings of the Royal Society B: Biological Sciences 268: 2473–2478. 10.1098/rspb.2001.1815 PMC1088902 11747566

[ecy70300-bib-0009] Brennan, A. , E. M. Hanks , J. A. Merkle , E. K. Cole , S. R. Dewey , A. B. Courtemanch , and P. C. Cross . 2018. “Examining Speed Versus Selection in Connectivity Models Using Elk Migration as an Example.” Landscape Ecology 33: 955–968.

[ecy70300-bib-0010] Buderman, F. E. , M. B. Hooten , J. S. Ivan , and T. M. Shenk . 2016. “A Functional Model for Characterizing Long‐Distance Movement Behaviour.” Methods in Ecology and Evolution 7: 264–273.

[ecy70300-bib-0011] Burt, W. H. 1943. “Territoriality and Home Range Concepts as Applied to Mammals.” Journal of Mammalogy 24: 346–352.

[ecy70300-bib-0012] Butler, M. J. , D. R. Stewart , G. M. Harris , M. T. Bidwell , and A. T. Pearse . 2022. “Space Use and Site Fidelity of Wintering Whooping Cranes on the Texas Gulf Coast.” Journal of Wildlife Management 86: e22226.

[ecy70300-bib-0013] Calabrese, J. M. , C. H. Fleming , and E. Gurarie . 2016. “Ctmm: An r Package for Analyzing Animal Relocation Data as a Continuous‐Time Stochastic Process.” Methods in Ecology and Evolution 7: 1124–1132.

[ecy70300-bib-0014] Calenge, C. 2006. “The Package “Adehabitat” for the R Software: A Tool for the Analysis of Space and Habitat Use by Animals.” Ecological Modelling 197: 516–519.

[ecy70300-bib-0015] Coe, P. K. , R. M. Nielson , D. H. Jackson , J. B. Cupples , N. E. Seidel , B. K. Johnson , S. C. Gregory , G. A. Bjornstrom , A. N. Larkins , and D. A. Speten . 2015. “Identifying Migration Corridors of Mule Deer Threatened by Highway Development.” Wildlife Society Bulletin 39: 256–267.

[ecy70300-bib-0016] Cohen, B. S. , T. J. Prebyl , B. A. Collier , and M. J. Chamberlain . 2018. “Home Range Estimator Method and GPS Sampling Schedule Affect Habitat Selection Inferences for Wild Turkeys.” Wildlife Society Bulletin 42: 150–159.

[ecy70300-bib-0017] Crane, M. , I. Silva , B. M. Marshall , and C. T. Strine . 2021. “Lots of Movement, Little Progress: A Review of Reptile Home Range Literature.” PeerJ 9: e11742. 34322323 10.7717/peerj.11742 PMC8300531

[ecy70300-bib-0018] Cumming, G. S. , and D. Cornélis . 2012. “Quantitative Comparison and Selection of Home Range Metrics for Telemetry Data.” Diversity and Distributions 18: 1057–1065.

[ecy70300-bib-0019] Dalke, P. D. , and P. R. Sime . 1938. “Home and Seasonal Ranges of the Eastern Cottontail in Connecticut.” Transcripts of the North American Wildlife Conference 3: 659–669.

[ecy70300-bib-0020] Dickie, M. , R. Serrouya , T. Avgar , P. McLoughlin , R. S. McNay , C. DeMars , S. Boutin , and A. T. Ford . 2022. “Resource Exploitation Efficiency Collapses the Home Range of an Apex Predator.” Ecology 103: e3642. 35066867 10.1002/ecy.3642

[ecy70300-bib-0021] Downs, J. A. , and M. W. Horner . 2008. “Effects of Point Pattern Shape on Home‐Range Estimates.” The Journal of Wildlife Management 72: 1813–1818.

[ecy70300-bib-0022] Dunn, J. E. , and P. S. Gipson . 1977. “Analysis of Radio Telemetry Data in Studies of Home Range.” Biometrics 33: 85–101.

[ecy70300-bib-0023] Eckrich, C. A. , P. K. Coe , D. A. Clark , R. M. Nielson , J. Lombardi , S. C. Gregory , M. J. Hedrick , B. K. Johnson , and D. H. Jackson . 2020. “Summer Habitat Use of Female Mule Deer in Oregon.” Journal of Wildlife Management 84: 576–587.

[ecy70300-bib-0024] Einstein, A. 1905. “Über die von der molekularkinetischen Theorie der Wärme geforderte Bewegung von in ruhenden Flüssigkeiten suspendierten Teilchen.” Annalen der Physik 4: 549–560.

[ecy70300-bib-0025] Farhadinia, M. S. , P. J. Johnson , D. W. Macdonald , and L. T. B. Hunter . 2018. “Anchoring and Adjusting Amidst Humans: Ranging Behavior of Persian Leopards along the Iran‐Turkmenistan Borderland.” PLoS One 13: e0196602. 29719005 10.1371/journal.pone.0196602 PMC5931651

[ecy70300-bib-0026] Fieberg, J. , and L. Börger . 2012. “Could you Please Phrase “Home Range” as a Question?” Journal of Mammalogy 93: 890–902.

[ecy70300-bib-0027] Find'o, S. , M. Skuban , M. Kajba , J. Chalmers , and M. Kalaš . 2018. “Identifying Attributes Associated with Brown Bear (*Ursus arctos*) Road‐Crossing and Roadkill Sites.” Canadian Journal of Zoology 97: 156–164.

[ecy70300-bib-0028] Fleming, C. H. , and J. M. Calabrese . 2017. “A New Kernel Density Estimator for Accurate Home‐Range and Species‐Range Area Estimation.” Methods in Ecology and Evolution 8: 571–579.

[ecy70300-bib-0029] Fleming, C. H. , J. M. Calabrese , T. Mueller , K. A. Olson , P. Leimgruber , and W. F. Fagan . 2014a. “From Fine‐Scale Foraging to Home Ranges: A Semivariance Approach to Identifying Movement Modes across Spatiotemporal Scales.” American Naturalist 183: E154–E167. 10.1086/675504 24739204

[ecy70300-bib-0030] Fleming, C. H. , J. M. Calabrese , T. Mueller , K. A. Olson , P. Leimgruber , and W. F. Fagan . 2014b. “Non‐Markovian Maximum Likelihood Estimation of Autocorrelated Movement Processes.” Methods in Ecology and Evolution 5: 462–472.

[ecy70300-bib-0031] Fleming, C. H. , W. F. Fagan , T. Mueller , K. A. Olson , P. Leimgruber , and J. M. Calabrese . 2015. “Rigorous Home Range Estimation with Movement Data: A New Autocorrelated Kernel Density Estimator.” Ecology 96: 1182–1188. 26236833 10.1890/14-2010.1

[ecy70300-bib-0032] Fleming, C. H. , W. F. Fagan , T. Mueller , K. A. Olson , P. Leimgruber , and J. M. Calabrese . 2016. “Estimating Where and How Animals Travel: An Optimal Framework for Path Reconstruction from Autocorrelated Tracking Data.” Ecology 97: 576–582. 27197385 10.1890/15-1607

[ecy70300-bib-0033] Fleming, C. H. , M. J. Noonan , E. P. Medici , and J. M. Calabrese . 2019. “Overcoming the Challenge of Small Effective Sample Sizes in Home‐Range Estimation.” Methods in Ecology and Evolution 10: 1679–1689.

[ecy70300-bib-0034] Fleming, C. H. , D. Sheldon , W. F. Fagan , P. Leimgruber , T. Mueller , D. Nandintsetseg , M. J. Noonan , et al. 2018. “Correcting for Missing and Irregular Data in Home‐Range Estimation.” Ecological Applications 28: 1003–1010. 29450936 10.1002/eap.1704

[ecy70300-bib-0035] Fleming, C. H. , Y. Subaşı , and J. M. Calabrese . 2015. “Maximum‐Entropy Description of Animal Movement.” Physical Review E 91: 032107. 10.1103/PhysRevE.91.032107 25871054

[ecy70300-bib-0036] Ford, A. T. , M. J. Noonan , K. Bollefer , R. Gill , C. Legebokow , and R. Serrouya . 2023. “The Effects of Maternal Penning on the Movement Ecology of Mountain Caribou.” Animal Conservation 26: 72–85.

[ecy70300-bib-0037] Gaston, K. J. , S. F. Jackson , L. Cantú‐Salazar , and G. Cruz‐Piñón . 2008. “The Ecological Performance of Protected Areas.” Annual Review of Ecology, Evolution, and Systematics 39: 93–113.

[ecy70300-bib-0038] Getz, W. M. , S. Fortmann‐Roe , P. C. Cross , A. J. Lyons , S. J. Ryan , and C. C. Wilmers . 2007. “LoCoH: Nonparameteric Kernel Methods for Constructing Home Ranges and Utilization Distributions.” PLoS One 2: e207. 17299587 10.1371/journal.pone.0000207 PMC1797616

[ecy70300-bib-0039] Getz, W. M. , and C. C. Wilmers . 2004. “A Local Nearest‐Neighbor Convex‐Hull Construction of Home Ranges and Utilization Distributions.” Ecography 27: 489–505.

[ecy70300-bib-0040] Gill, R. , R. Serrouya , A. M. Calvert , A. Ford , R. Steenweg , and M. J. Noonan . 2023. “Movement Ecology of Endangered Caribou during a COVID‐19 Mediated Pause in Winter Recreation.” Animal Conservation 27: 350–363.

[ecy70300-bib-0041] Gupte, P. R. , C. E. Beardsworth , O. Spiegel , E. Lourie , S. Toledo , R. Nathan , and A. I. Bijleveld . 2022. “A Guide to Pre‐Processing High‐Throughput Animal Tracking Data.” Journal of Animal Ecology 91: 287–307. 34657296 10.1111/1365-2656.13610 PMC9299236

[ecy70300-bib-0042] Gurarie, E. , C. H. Fleming , W. F. Fagan , K. L. Laidre , J. Hernández‐Pliego , and O. Ovaskainen . 2017. “Correlated Velocity Models as a Fundamental Unit of Animal Movement: Synthesis and Applications.” Movement Ecology 5: 13. 28496983 10.1186/s40462-017-0103-3 PMC5424322

[ecy70300-bib-0043] Gurarie, E. , and O. Ovaskainen . 2011. “Characteristic Spatial and Temporal Scales Unify Models of Animal Movement.” American Naturalist 178: 113–123. 10.1086/660285 21670582

[ecy70300-bib-0044] Hanks, E. M. , M. B. Hooten , and M. W. Alldredge . 2015. “Continuous‐Time Discrete‐Space Models for Animal Movement.” The Annals of Applied Statistics 9: 145–165.

[ecy70300-bib-0045] Heit, D. R. , W. Ortiz‐Calo , and R. A. Montgomery . 2021. “Landscape Complexity Persists as a Critical Source of Bias in Terrestrial Animal Home Range Estimation.” Ecology 102: e03427. 34105787 10.1002/ecy.3427

[ecy70300-bib-0046] Hooker, M. , D. Jared , R. Warren , K. Miller , and M. Chamberlain . 2020. “Characterizing American Black Bear (*Ursus americanus*) Highway Crossing Locations in Central Georgia.” Journal of the Southeastern Association of Fish and Wildlife Agencies 7: 227–237.

[ecy70300-bib-0047] Hooten, M. B. , and D. S. Johnson . 2017. “Basis Function Models for Animal Movement.” Journal of the American Statistical Association 112: 578–589.

[ecy70300-bib-0048] Horne, J. S. , J. Fieberg , L. Börger , J. L. Rachlow , J. M. Calabrese , and C. H. Fleming . 2020. “Animal Home Ranges: Concepts, Uses, and Estimation.” In Population Ecology in Practice 315–332. Oxford: Wiley‐Blackwell.

[ecy70300-bib-0049] Horne, J. S. , E. O. Garton , S. M. Krone , and J. S. Lewis . 2007. “Analyzing Animal Movements Using Brownian Bridges.” Ecology 88: 2354–2363. 17918412 10.1890/06-0957.1

[ecy70300-bib-0050] Hurvich, C. M. , and C. L. Tsai . 1989. “Regression and Time Series Model Selection in Small Samples.” Biometrika 76: 297–307.

[ecy70300-bib-0051] Jeltsch, F. , D. Bonte , G. Pe'er , B. Reineking , P. Leimgruber , N. Balkenhol , B. Schröder , et al. 2013. “Integrating Movement Ecology with Biodiversity Research ‐ Exploring New Avenues to Address Spatiotemporal Biodiversity Dynamics.” Movement Ecology 1: 6. 25709820 10.1186/2051-3933-1-6 PMC4337763

[ecy70300-bib-0052] Jennrich, R. I. , and F. B. Turner . 1969. “Measurement of Non‐circular Home Range.” Journal of Theoretical Biology 22: 227–237. 5783911 10.1016/0022-5193(69)90002-2

[ecy70300-bib-0053] Johnson, D. S. , J. M. London , and C. E. Kuhn . 2011. “Bayesian Inference for Animal Space Use and Other Movement Metrics.” Journal of Agricultural, Biological, and Environmental Statistics 16: 357–370.

[ecy70300-bib-0054] Johnson, D. S. , J. M. London , M. A. Lea , and J. W. Durban . 2008. “Continuous‐Time Correlated Random Walk Model for Animal Telemetry Data.” Ecology 89: 1208–1215. 18543615 10.1890/07-1032.1

[ecy70300-bib-0055] Johnston, A. N. , W. M. V. Haegen , and S. D. West . 2020. “Differential Resource Use between Native and Introduced Gray Squirrels.” Journal of Wildlife Management 84: 726–738.

[ecy70300-bib-0056] Kareiva, P. M. , and N. Shigesada . 1983. “Analyzing Insect Movement as a Correlated Random Walk.” Oecologia 56: 234–238. 28310199 10.1007/BF00379695

[ecy70300-bib-0057] Kays, R. , M. C. Crofoot , W. Jetz , and M. Wikelski . 2015. “Terrestrial Animal Tracking as an Eye on Life and Planet.” Science 348: aaa2478. 26068858 10.1126/science.aaa2478

[ecy70300-bib-0058] Keith, J. M. , D. Spring , and T. Kompas . 2019. “Delimiting a Species' Geographic Range Using Posterior Sampling and Computational Geometry.” Scientific Reports 9: 8938. 31222114 10.1038/s41598-019-45318-5 PMC6586837

[ecy70300-bib-0059] Kie, J. G. 2013. “A Rule‐Based Ad Hoc Method for Selecting a Bandwidth in Kernel Home‐Range Analyses.” Animal Biotelemetry 1: 13.

[ecy70300-bib-0060] Kie, J. G. , J. Matthiopoulos , J. Fieberg , R. A. Powell , F. Cagnacci , M. S. Mitchell , J. M. Gaillard , and P. R. Moorcroft . 2010. “The Home‐Range Concept: Are Traditional Estimators Still Relevant with Modern Telemetry Technology?” Philosophical Transactions of the Royal Society B: Biological Sciences 365: 2221–2231. 10.1098/rstb.2010.0093 PMC2894967 20566499

[ecy70300-bib-0061] Koizumi, C. L. , and A. E. Derocher . 2019. “Predation Risk and Space Use of a Declining Dall Sheep (*Ovis dalli dalli*) Population.” PLoS One 14: e0215519. 30986250 10.1371/journal.pone.0215519 PMC6464218

[ecy70300-bib-0062] Kranstauber, B. , R. Kays , S. D. LaPoint , M. Wikelski , and K. Safi . 2012. “A Dynamic Brownian Bridge Movement Model to Estimate Utilization Distributions for Heterogeneous Animal Movement.” Journal of Animal Ecology 81: 738–746. 22348740 10.1111/j.1365-2656.2012.01955.x

[ecy70300-bib-0063] Langrock, R. , R. King , J. Matthiopoulos , L. Thomas , D. Fortin , and J. M. Morales . 2012. “Flexible and Practical Modeling of Animal Telemetry Data: Hidden Markov Models and Extensions.” Ecology 93: 2336–2342. 23236905 10.1890/11-2241.1

[ecy70300-bib-0064] Laver, P. N. , and M. J. Kelly . 2008. “A Critical Review of Home Range Studies.” Journal of Wildlife Management 72: 290–298.

[ecy70300-bib-0065] Lyons, A. J. , W. C. Turner , and W. M. Getz . 2013. “Home Range plus: A Space‐Time Characterization of Movement over Real Landscapes.” Movement Ecology 1: 2. 25709816 10.1186/2051-3933-1-2 PMC4337754

[ecy70300-bib-0066] Manly, B. , L. McDonald , D. Thomas , T. McDonald , and W. Erickson . 2007. Resource Selection by Animals: Statistical Design and Analysis for Field Studies. Dordrecht: Springer Science & Business Media.

[ecy70300-bib-0067] Marques, A. T. , C. D. Santos , F. Hanssen , A. R. Muñoz , A. Onrubia , M. Wikelski , F. Moreira , J. M. Palmeirim , and J. P. Silva . 2020. “Wind Turbines Cause Functional Habitat Loss for Migratory Soaring Birds.” Journal of Animal Ecology 89: 93–103. 30762229 10.1111/1365-2656.12961

[ecy70300-bib-0068] Martins, C. C. A. , A. Andriolo , M. H. Engel , P. G. Kinas , and C. H. Saito . 2013. “Identifying Priority Areas for Humpback Whale Conservation at Eastern Brazilian Coast.” Ocean & Coastal Management 75: 63–71.

[ecy70300-bib-0069] Marzluff, J. M. , J. J. Millspaugh , P. Hurvitz , and M. S. Handcock . 2004. “Relating Resources to a Probabilistic Measure of Space Use: Forest Fragments and Steller's Jays.” Ecology 85: 1411–1427.

[ecy70300-bib-0070] Meyer, N. F. V. , J. P. King , M. Mahony , J. Clulow , C. Beranek , C. Reedman , N. Balkenhol , and M. W. Hayward . 2021. “Large Area Used by Squirrel Gliders in an Urban Area, Uncovered Using GPS Telemetry.” Ecology and Evolution 11: 7147–7153. 34188802 10.1002/ece3.7644 PMC8216951

[ecy70300-bib-0071] Michelot, T. , P. G. Blackwell , and J. Matthiopoulos . 2019. “Linking Resource Selection and Step Selection Models for Habitat Preferences in Animals.” Ecology 100: e02452. 30047993 10.1002/ecy.2452

[ecy70300-bib-0072] Michelot, T. , P. Gloaguen , P. G. Blackwell , and M. P. Étienne . 2019. “The Langevin Diffusion as a Continuous‐Time Model of Animal Movement and Habitat Selection.” Methods in Ecology and Evolution 10: 1894–1907.

[ecy70300-bib-0073] Millspaugh, J. J. , R. M. Nielson , L. McDonald , J. M. Marzluff , R. A. Gitzen , C. D. Rittenhouse , M. W. Hubbard , and S. L. Sheriff . 2006. “Analysis of Resource Selection Using Utilization Distributions.” Journal of Wildlife Management 70: 384–395.

[ecy70300-bib-0074] Mirski, P. , Z. Cenian , M. Dagys , S. Daróczi , D. Dementavičius , G. Maciorowski , S. Menderski , et al. 2021. “Sex‐, Landscape‐ and Climate‐Dependent Patterns of Home‐Range Size – A Macroscale Study on an Avian Generalist Predator.” Ibis 163: 641–657.

[ecy70300-bib-0075] Moorcroft, P. R. , M. A. Lewis , and R. L. Crabtree . 2006. “Mechanistic Home Range Models Capture Spatial Patterns and Dynamics of Coyote Territories in Yellowstone.” Proceedings of the Royal Society B: Biological Sciences 273: 1651–1659. 10.1098/rspb.2005.3439 PMC1704082 16769637

[ecy70300-bib-0076] Morales, J. M. , D. T. Haydon , J. Frair , K. E. Holsinger , and J. M. Fryxell . 2004. “Extracting More out of Relocation Data: Building Movement Models as Mixtures of Random Walks.” Ecology 85: 2436–2445.

[ecy70300-bib-0077] Morato, R. G. , J. J. Thompson , A. Paviolo , J. A. de La Torre , F. Lima , R. T. McBride, Jr. , R. C. Paula , et al. 2018. “Jaguar Movement Database: a GPS‐Based Movement Dataset of An Apex Predator in the Neotropics.” Ecology 99: 1691. 10.1002/ecy.2379. 29961270

[ecy70300-bib-0078] Moriarty, K. M. , M. A. Linnell , B. E. Chasco , C. W. Epps , and W. J. Zielinski . 2017. “Using High‐Resolution Short‐Term Location Data to Describe Territoriality in Pacific Martens.” Journal of Mammalogy 98: 679–689.

[ecy70300-bib-0079] Nathan, R. , W. M. Getz , E. Revilla , M. Holyoak , R. Kadmon , D. Saltz , and P. E. Smouse . 2008. “A Movement Ecology Paradigm for Unifying Organismal Movement Research.” Proceedings of the National Academy of Sciences 105: 19052–19059. 10.1073/pnas.0800375105 PMC2614714 19060196

[ecy70300-bib-0080] Nathan, R. , C. T. Monk , R. Arlinghaus , T. Adam , J. Alós , M. Assaf , H. Baktoft , et al. 2022. “Big‐Data Approaches Lead to an Increased Understanding of the Ecology of Animal Movement.” Science 375: eabg1780. 35175823 10.1126/science.abg1780

[ecy70300-bib-0081] Nielson, R. M. , H. Sawyer , and T. L. McDonald . 2013. “BBMM: Brownian Bridge Movement Model for Estimating the Movement Path of an Animal Using Discrete Location Data.” http://cran.r-project.org/package=BBMM.

[ecy70300-bib-0082] Noonan, M. J. , C. H. Fleming , M. A. Tucker , R. Kays , A. L. Harrison , M. C. Crofoot , B. Abrahms , et al. 2020. “Effects of Body Size on Estimation of Mammalian Area Requirements.” Conservation Biology 34: 1017–1028. 32362060 10.1111/cobi.13495 PMC7496598

[ecy70300-bib-0083] Noonan, M. J. , R. Martinez‐Garcia , G. H. Davis , M. C. Crofoot , R. Kays , B. T. Hirsch , D. Caillaud , et al. 2021. “Estimating Encounter Location Distributions from Animal Tracking Data.” Methods in Ecology and Evolution 12: 1158–1173.

[ecy70300-bib-0084] Noonan, M. J. , C. Newman , A. Markham , K. Bilham , C. D. Buesching , and D. W. Macdonald . 2018. “In Situ Behavioral Plasticity as Compensation for Weather Variability: Implications for Future Climate Change.” Climatic Change 149: 457–471.

[ecy70300-bib-0085] Noonan, M. J. , M. A. Tucker , C. H. Fleming , T. S. Akre , S. C. Alberts , A. H. Ali , J. Altmann , et al. 2019. “A Comprehensive Analysis of Autocorrelation and Bias in Home Range Estimation.” Ecological Monographs 89: e01344.

[ecy70300-bib-0086] Pagès, J. F. , S. R. Jenkins , T. J. Bouma , E. Sharps , and M. W. Skov . 2019. “Opposing Indirect Effects of Domestic Herbivores on Saltmarsh Erosion.” Ecosystems 22: 1055–1068.

[ecy70300-bib-0087] Papageorgiou, D. , and D. R. Farine . 2020. “Group Size and Composition Influence Collective Movement in a Highly Social Terrestrial Bird.” eLife 9: e59902. 33168135 10.7554/eLife.59902 PMC7655099

[ecy70300-bib-0088] Pe'er, G. , M. A. Tsianou , K. W. Franz , Y. G. Matsinos , A. D. Mazaris , D. Storch , L. Kopsova , et al. 2014. “Toward Better Application of Minimum Area Requirements in Conservation Planning.” Biological Conservation 170: 92–102.

[ecy70300-bib-0089] Péron, G. , C. H. Fleming , R. C.d. Paula , N. Mitchell , M. Strohbach , P. Leimgruber , and J. M. Calabrese . 2017. “Periodic Continuous‐Time Movement Models Uncover Behavioral Changes of Wild Canids along Anthropization Gradients.” Ecological Monographs 87: 442–456.

[ecy70300-bib-0090] Petroelje, T. R. , T. M. Kautz , D. E. Beyer , and J. L. Belant . 2021. “Interference Competition between Wolves and Coyotes during Variable Prey Abundance.” Ecology and Evolution 11: 1413–1431. 33598141 10.1002/ece3.7153 PMC7863399

[ecy70300-bib-0091] Prince, A. , M. C. Chitwood , M. A. Lashley , C. S. DePerno , and C. E. Moorman . 2016. “Resource Selection by Southeastern Fox Squirrels in a Fire‐Maintained Forest System.” Journal of Mammalogy 97: 631–638.

[ecy70300-bib-0092] R Core Team . 2020. “R: A Language and Environment for Statistical Computing.” R Foundation for Statistical Computing, Vienna, Austria. https://www.r-project.org/.

[ecy70300-bib-0093] Rechetelo, J. , A. Grice , A. E. Reside , B. D. Hardesty , and J. Moloney . 2016. “Movement Patterns, Home Range Size and Habitat Selection of an Endangered Resource Tracking Species, the Black‐Throated Finch (*Poephila cincta cincta*).” PLoS One 11: e0167254. 27902764 10.1371/journal.pone.0167254 PMC5130248

[ecy70300-bib-0094] Sawyer, H. , M. J. Kauffman , R. M. Nielson , and J. S. Horne . 2009. “Identifying and Prioritizing Ungulate Migration Routes for Landscape‐Level Conservation.” Ecological Applications 19: 2016–2025. 20014575 10.1890/08-2034.1

[ecy70300-bib-0095] Sawyer, H. , J. A. Merkle , A. D. Middleton , S. P. H. Dwinnell , and K. L. Monteith . 2019. “Migratory Plasticity Is Not Ubiquitous among Large Herbivores.” Journal of Animal Ecology 88: 450–460. 30449042 10.1111/1365-2656.12926

[ecy70300-bib-0096] Scharf, A. K. , J. L. Belant , D. E. Beyer , M. Wikelski , and K. Safi . 2018. “Habitat Suitability Does Not Capture the Essence of Animal‐Defined Corridors.” Movement Ecology 6: 18. 30275955 10.1186/s40462-018-0136-2 PMC6158861

[ecy70300-bib-0097] Scharf, H. , M. B. Hooten , and D. S. Johnson . 2017. “Imputation Approaches for Animal Movement Modeling.” Journal of Agricultural, Biological, and Environmental Statistics 22: 335–352.

[ecy70300-bib-0098] Schick, R. S. , S. R. Loarie , F. Colchero , B. D. Best , A. Boustany , D. A. Conde , P. N. Halpin , L. N. Joppa , C. M. McClellan , and J. S. Clark . 2008. “Understanding Movement Data and Movement Processes: Current and Emerging Directions.” Ecology Letters 11: 1338–1350. 19046362 10.1111/j.1461-0248.2008.01249.x

[ecy70300-bib-0099] Schlägel, U. E. , J. Signer , A. Herde , S. Eden , F. Jeltsch , J. A. Eccard , and M. Dammhahn . 2019. “Estimating Interactions between Individuals from Concurrent Animal Movements.” Methods in Ecology and Evolution 10: 1234–1245.

[ecy70300-bib-0100] Signer, J. , J. Fieberg , and T. Avgar . 2019. “Animal Movement Tools (Amt): R Package for Managing Tracking Data and Conducting Habitat Selection Analyses.” Ecology and Evolution 9: 880–890. 30766677 10.1002/ece3.4823 PMC6362447

[ecy70300-bib-0101] Signer, J. , and J. R. Fieberg . 2021. “A Fresh Look at an Old Concept: Home‐Range Estimation in a Tidy World.” PeerJ 9: e11031. 33954027 10.7717/peerj.11031 PMC8048401

[ecy70300-bib-0102] Silva, I. , M. Crane , B. M. Marshall , and C. T. Strine . 2020. “Reptiles on the Wrong Track? Moving beyond Traditional Estimators with Dynamic Brownian Bridge Movement Models.” Movement Ecology 8: 43. 33133609 10.1186/s40462-020-00229-3 PMC7592577

[ecy70300-bib-0103] Silva, I. , C. H. Fleming , M. J. Noonan , J. Alston , C. Folta , W. F. Fagan , and J. M. Calabrese . 2022. “Autocorrelation‐Informed Home Range Estimation: A Review and Practical Guide.” Methods in Ecology and Evolution 13: 534–544.

[ecy70300-bib-0104] Snider, M. H. , V. R. Athreya , G. A. Balme , L. R. Bidner , M. S. Farhadinia , J. Fattebert , M. E. Gompper , et al. 2021. “Home Range Variation in Leopards Living across the Human Density Gradient.” Journal of Mammalogy 102: 1138–1148.

[ecy70300-bib-0105] Sutherland, W. J. , R. P. Freckleton , H. C. J. Godfray , S. R. Beissinger , T. Benton , D. D. Cameron , Y. Carmel , et al. 2013. “Identification of 100 fundamental ecological questions.” Journal of Ecology 101: 58–67.

[ecy70300-bib-0106] Tédonzong, L. R. D. , J. Willie , A. M. P. Keuko , J. K. Kuenbou , G. Njotah , M. N. Tchamba , N. Tagg , and L. Lens . 2018. “Using Abundance and Habitat Variables to Identify High Conservation Value Areas for Threatened Mammals.” Biodiversity and Conservation 27: 1115–1137.

[ecy70300-bib-0107] Uhlenbeck, G. E. , and L. S. Ornstein . 1930. “On the Theory of the Brownian Motion.” Physical Review 36: 823–841.

[ecy70300-bib-0108] van Eeden, L. M. , M. S. Crowther , C. R. Dickman , D. W. Macdonald , W. J. Ripple , E. G. Ritchie , and T. M. Newsome . 2018. “Managing Conflict between Large Carnivores and Livestock.” Conservation Biology 32: 26–34. 28556528 10.1111/cobi.12959

[ecy70300-bib-0109] Walter, W. D. , D. P. Onorato , and J. W. Fischer . 2015. “Is there a Single Best Estimator? Selection of Home Range Estimators Using Area‐under‐the‐Curve.” Movement Ecology 3: 10. 25973204 10.1186/s40462-015-0039-4 PMC4429481

[ecy70300-bib-0110] Whitehead, H. , and I. D. Jonsen . 2013. “Inferring Animal Densities from Tracking Data Using Markov Chains.” PLoS One 8: e60901. 23630574 10.1371/journal.pone.0060901 PMC3632542

[ecy70300-bib-0111] Wilson, K. , E. Hanks , and D. Johnson . 2018. “Estimating Animal Utilization Densities Using Continuous‐Time Markov Chain Models.” Methods in Ecology and Evolution 9: 1232–1240.

[ecy70300-bib-0112] Winder, V. L. , L. B. McNew , J. C. Pitman , and B. K. Sandercock . 2017. “Space Use of Female Greater Prairie‐Chickens in Response to Fire and Grazing Interactions.” Rangeland Ecology & Management 70: 165–174.

[ecy70300-bib-0113] Worton, B. J. 1989. “Kernel Methods for Estimating the Utilization Distribution in Home‐Range Studies.” Ecology 70: 164–168.

[ecy70300-bib-0114] Zeller, K. A. , D. W. Wattles , and S. DeStefano . 2018. “Incorporating Road Crossing Data into Vehicle Collision Risk Models for Moose (*Alces americanus*) in Massachusetts, USA.” Environmental Management 62: 518–528. 29744581 10.1007/s00267-018-1058-x

