# Supplementary material for: Clarifying space use concepts in ecology: Range vs. occurrence distributions
Source: Ecology. 2026 Mar 7;107(3):e70300. doi: 10.1002/ecy.70300 (PMC12966954; doi:10.1002/ecy.70300)
Supplement: Supplementary file 1 — Appendix S1. [file ECY-107-e70300-s001.pdf]

Appendix S1

Clarifying space use concepts in ecology: Range vs. occurrence distributions

Jesse M. Alston, Christen H. Fleming, Michael J. Noonan, Marlee A. Tucker, Inês Silva, Cody Folta, Thomas S.B. Akre, Abdullahi H. Ali, Jerrold L. Belant, Dean Beyer, Niels Blaum, Katrin Böhning-Gaese, Rogério Cunha de Paula, Jasja Dekker, Jonathan Drescher-Lehman, Nina Farwig, Claudia Fichtel, Christina Fischer, Adam T. Ford, René Janssen, Florian Jeltsch, Peter M. Kappeler, Scott D. LaPoint, A. Catherine Markham, E. Patricia Medici, Ronaldo Gonçalves Morato, Ran Nathan, Kirk A. Olson, Bruce D. Patterson, Tyler R. Petroelje, Emiliano Esterci Ramalho, Sascha Rösner, Luiz Gustavo Rodrigues Oliveira-Santos, Dana G. Schabo, Nuria Selva, Agnieszka Sergiel, Orr Spiegel, Wiebke Ullmann, Filip Zi˛eba, Tomasz Zw¼acz-Kozica, George Wittemyer, William F. Fagan, Thomas Müller, and Justin M. Calabrese

Ecology

Table S1. *Movebank identifiers and URLs for data sets used in empirical analyses in this paper that are not included in the Open Research Statement.*

Species	Identifier	URL
Canis latrans	8159699	https://www.movebank.org/cms/webapp?gwt_fragment=page=studies,path=study8159699
Canis lupus	8159399	https://www.movebank.org/cms/webapp?gwt_fragment=page=studies,path=study8159399
Chrysocyon brachyurus	18156143	https://www.movebank.org/cms/webapp?gwt_fragment=page=studies,path=study18156143
Felis silvestris	40386102	https://www.movebank.org/cms/webapp?gwt_fragment=page=studies,path=study40386102
Gyps africanus	2919708	https://www.movebank.org/cms/webapp?gwt_fragment=page=studies,path=study2919708
Lepus europaeus	25727477	https://www.movebank.org/cms/webapp?gwt_fragment=page=studies,path=study25727477
Martes pennanti	2964494	https://www.movebank.org/cms/webapp?gwt_fragment=page=studies,path=study2964494
Panthera leo	220229	https://www.movebank.org/cms/webapp?gwt_fragment=page=studies,path=study220229
Papio cynocephalus	222027	https://www.movebank.org/cms/webapp?gwt_fragment=page=studies,path=study222027
Syncerus caffer	1764627	https://www.movebank.org/cms/webapp?gwt_fragment=page=studies,path=study1764627
Tapirus terrestris	1907973121	https://www.movebank.org/cms/webapp?gwt_fragment=page=studies,path=study1907973121
Torgos tracheliotus	2919708	https://www.movebank.org/cms/webapp?gwt_fragment=page=studies,path=study2919708
Ursus americanus	8170674	https://www.movebank.org/cms/webapp?gwt_fragment=page=studies,path=study8170674
